# Somatostatin Analogs Versus Active Surveillance in Small Pancreatic Neuroendocrine Tumors

**DOI:** 10.1097/MPA.0000000000002425

**Published:** 2024-11-28

**Authors:** Maria Grazia Maratta, Sabrina Chiloiro, Salvatore Raia, Brigida A. Maiorano, Guido Horn, Maria Gabriella Brizi, Vittoria Rufini, Romina Grazia Giancipoli, Laura De Marinis, Antonio Bianchi, Alfredo Pontecorvi, Giovanni Schinzari, Frediano Inzani, Giampaolo Tortora, Guido Rindi

**Affiliations:** * Fondazione Policlinico Universitario Agostino Gemelli - IRCCS - Università Cattolica del Sacro Cuore European NeuroEndocrine Tumor Society (ENETS) Center of Excellence for the Diagnosis and Cure of Neuroendocrine Tumors, Rome, Italy; † Comprehensive Cancer Center, Fondazione Policlinico Universitario Agostino Gemelli, IRCCS - Università Cattolica del Sacro Cuore, Rome, Italy; ‡ Division of Endocrinology and Metabolism, Fondazione Policlinico Universitario A. Gemelli, IRCCS - Università Cattolica del Sacro Cuore, Rome, Italy; § Department of Radiology, Fondazione Policlinico Universitario Agostino Gemelli, IRCCS - Università Cattolica del Sacro Cuore, Rome, Italy; ∥ Section of Nuclear Medicine, Department of Radiological Sciences, Radiotherapy and Haematology, Università Cattolica del Sacro Cuore, Rome, Italy; ¶ Nuclear Medicine Unit, Department of Radiology, Radiotherapy and Haematology, Fondazione Policlinico Universitario Agostino Gemelli IRCCS, Rome Italy; # Anatomic Pathology Section, Department of Life Sciences and Public Health, Università Cattolica del Sacro Cuore, Largo A. Gemelli, 8, Rome, Italy; ** Anatomic Pathology Unit, Department of Sciences of Woman and Child Health and Public Health, Fondazione Policlinico Universitario A. Gemelli, IRCCS, Rome, Italy

**Keywords:** neuroendocrine cancer, pancreatic cancer, PanNET, NET, somatostatin analogs, efficacy, surveillance

## Abstract

**Objectives:**

The best strategy for nonfunctioning, sporadic, G1-G2 pancreatic neuroendocrine tumors ≤2 cm is unknown. An active surveillance is usually recommended. The PROMID and the CLARINET studies proved the value of somatostatin analog (SSA) treatment in advanced gastro-entero-pancreatic neuroendocrine tumors. The aim of this study is to assess the value of SSA in pancreatic NET (PanNET) ≤ 2 cm.

**Materials and Methods:**

We retrospectively collected data from 72 patients with sporadic nonfunctioning G1-G2 PanNETs ≤ 2 cm, which were either treated with somatostatin analogs (n = 31) or underwent active surveillance (n = 41) at our institution.

**Results:**

At a median follow-up of 53.7 months, the median progression-free survival was not reached in the treatment group versus an estimated progression-free survival of 85 months in the control group (hazard ratio, 0.11; *P* = 0.01), with a rate of progression or death up to 21.9% in the active surveillance group. Additionally, in the group of patients treated with somatostatin analogs, the response rate was 16.1% with 1 complete response.

**Conclusions:**

Our monocentric experience demonstrated a significant antiproliferative activity of somatostatin analogs in patients with sporadic, nonfunctionating G1-G2 PanNETs ≤ 2-cm delaying tumor progression and distant spread in small lesions that sometimes may reveal unpredictable aggressiveness.

Neuroendocrine tumors (NETs) are well differentiated neuroendocrine neoplasms thought to originate from cells of the diffuse neuroendocrine system.^1^ One of the most frequent sites of insurgence is the gastro-entero-pancreatic tract and comprises pancreatic NETs (PanNETs).^2^ In the last 3 decades the worldwide, incidence of PanNETs has been steadily increasing, possibly because of earlier detection by improved diagnostics.^3–5^ The largest fraction is made by nonfunctioning (NF) sporadic PanNETs.^6,7^ For advanced NF PanNETs, surgery and/or somatostatin analog (SSA) treatment are consistently offered to patients by current guidelines.^2^ However, for localized disease, the variable natural history, clinical symptoms, and prognosis make challenging the definition of standard management. Two main therapeutic options are indicated as acceptable by current guidelines and include intent-to-cure surgery or surveillance, both based on case-per-case NET multidisciplinary tumor board (MTB) decision.^2,8,9^ NF, sporadic G1-G2 PanNETs usually display an indolent course and an active surveillance (AS) strategy is considered safe in asymptomatic patients with ≤2 cm by the European Neuroendocrine Tumor Society guidelines.^9^ However, no evidence of the value of SSA treatment exists in such patient population. By converse, the antiproliferative effect of SSA therapy was proved in advanced GEP-NETs by both the PROMID and the CLARINET studies.^10–12^ The aim of this study is to assess the clinical value of SSA treatment in patients with sporadic, NF, G1-G2 PanNETs ≤2 cm.

## MATERIALS AND METHODS

### Study Design and Population

This work applied the Reporting of studies Conducted using Observational Routinely-collected health Data statement.^13^ In our retrospective observational study, we collected data from patients with sporadic, NF, well-differentiated G1-G2 PanNETs ≤ 2 cm referred to our center between June 2007 and June 2022. Data were collected from patients' medical records. All patients were age 18 years or older with a new histologically confirmed diagnosis of PanNETs, tumor size ≤2 cm at contrast enhanced computed tomography (CT) or magnetic resonance imaging (MRI). Patients with functional PanNETs including carcinoid syndrome were excluded as well as patients with a diagnosed heritable condition such as multiple endocrine neoplasia 1 (MEN-1) or Von Hippel–Lindau (VHL) or other. Mixed neuroendocrine/nonendocrine neoplasms (MiNEN) cases were also excluded. Every patient underwent a complete imaging workup including a high-quality cross-sectional imaging study, either CT or MRI of upper abdomen to define tumor size and stage. At least one ^68^Ga-SSA (^68^Ga-DOTANOC or ^68^Ga-DOTATOC) positron emission tomography/computed tomography scan performed at diagnosis or during the follow-up period was considered mandatory for complete staging to be enrolled in the study. All patients underwent endoscopic ultrasound endoscopy with tissue acquisition for histopathology analysis.^14^ The histological confirmation of PanNET was obtained for all patients and diagnosis and grading performed according to the World Health Organization 2019 criteria^15^; a histological second look was performed in all patients included in the study that were diagnosed before the current classification came into clinical practice. Somatostatin receptor (SSTR) expression was proved either *in vitro* by immunohistochemistry (IHC) on tissue according to the Volante score (SSTR2 only)^16^ or *in vivo* by ^68^Ga-SSA PET/CT according to the Krenning score (SSTR2, 5).^17^ No patient received any previous therapy for the disease. Patients were deemed eligible for AS or surgery following case-by-case discussion at our MTB and according to patient wish. We included in the analysis all patients who were excluded from a surgical approach due to age, comorbidity and high-risk of surgical complication, and those who refused surgery. In these cases, and to whom declined an AS only program, according to physician judgment and patient's choice, the option of SSA therapy (octreotide LAR 30 mg or lanreotide 120 mg every 28 days, on physician's choice) for disease control^10,12^ was offered additionally to the same workup of the AS program. For those enrolled in the AS, the follow-up strategy included a 6-month interval imaging follow-up with CT or MRI. Tumor assessments after baseline were performed by experienced radiologist according to the Response Evaluation Criteria in Solid Tumors 1.1 (RECIST 1.1) criteria.^18^ We considered progressive disease (PD) a 20% increase in largest diameter of the pancreatic lesions, conforming to RECIST 1.1 criteria or the increase over the limit of 2 cm or the appearance of 1 or more new lesions. All patients underwent follow-up CT/MRI imaging at 6-month intervals according to current recommendation.^2^ All patients provided written informed consent to treatment, medical procedures, and data collection. All the procedures were conducted in accordance with the precepts of good clinical practice. The study follows the ethical standards of the Helsinki Declaration.

### Endpoints

The primary endpoint was disease progression-free survival (PFS) defined as the time from the start of treatment (SSA or AS) to radiological evidence of progression disease or death. Secondary endpoints were overall response rate (RR) defined as the percentage of people in each treatment groups who have radiological evidence of partial or complete response and overall survival (OS) defined as the time from diagnosis to overall (any cause) and disease-related death or latest follow-up. An exploratory analysis was performed to evaluate the survival impact of demographic and clinicopathological variables including age, sex, tumor size, grade Ki67 index, and SSTR2 expression by IHC.

### Statistical Analysis

Statistical analyses were performed using SPSS software (SPSS for Windows Version 29.0; SPSS Inc, Chicago, IL). Graphics were created using GraphPad Prism software (GraphPad Prism software for Windows Version 9.0.0, Dotmatics San Diego, CA). Baseline characteristics were expressed as counts and percentages if categorical, means with standard deviation if continuous and normal or median with interquartile range if continuous not normal. Normality was checked using the Shapiro-Wilk equation. Differences in categorical variables were tested using the chi-squared test while differences in continuous variables were assessed by the Student T test or Mann-Whitney test according to normality. Survival was described by the Kaplan-Meier method with Mantel-Haenszel log-rank test. Cox-regression analysis was used to assess, both singularly and collectively, the weight of clinically relevant covariates on survival outcomes. Statistical significance was declared at 2-sided *P* < 0.05.

## RESULTS

### Demographic and Baseline Characteristics of Patients

**FIGURE 1:**
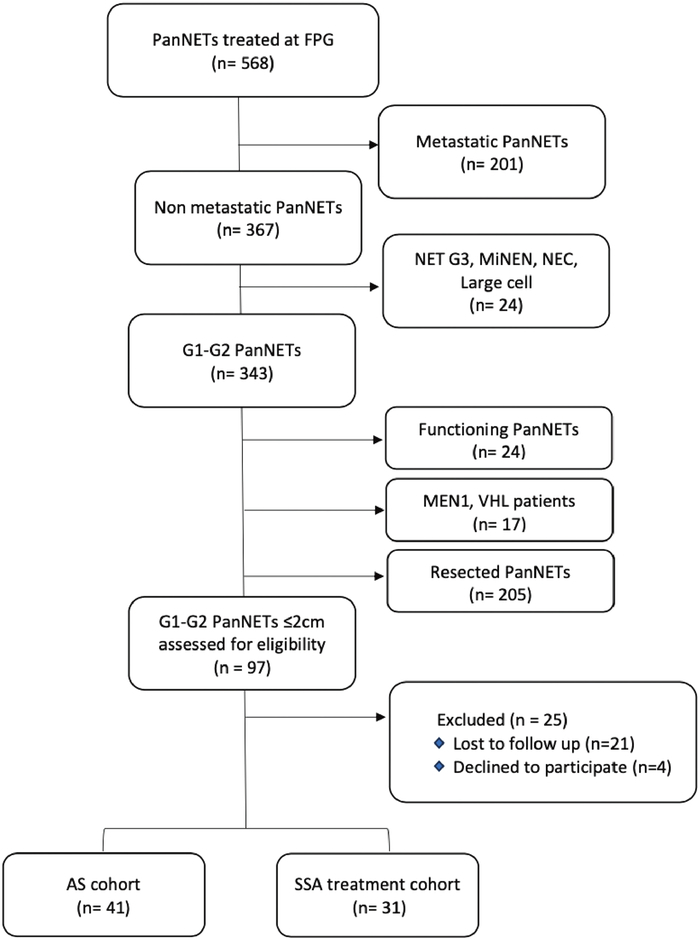
Data collection flowchart (CONSORT diagram). FPG, Fondazione Policlinico Universitario Agostino Gemelli - IRCCS; FUP, follow-up.

Table 1 reports the main clinical-pathological characteristics of this cohort. In 16 years, from 2007 to 2022, 568 patients affected by pancreatic NETs were referred to our Institution: 201 were metastatic at diagnosis; 11 had a mixed neuroendocrine/nonendocrine neoplasm; 13 had a high-grade neoplasm (NET G3/NEC/large-cell NET); 24 presented with functioning sporadic pancreatic tumors; 17 patients have a hereditary syndrome (15 MEN1, 2 Von Hippel–Lindau); 205 underwent surgery; 25 were lost to follow-up or declined to participate. All these cases were excluded from the current analysis (Fig. 1). Data included in this study were collected from 72 patients, 36 males and 36 females, with a new diagnosis of PanNET ≤ 2 cm. Patients' age at diagnosis ranged between 31–89 years, with a median age of 64 years. All patients were discussed at our MTB. According to current recommendations and the collaborative national guidelines by the Italian Association of Medical Oncology and Italian Association for Neuroendocrine Tumors^19^ an AS strategy was proposed to all patients. The ones who declined an AS program were proposed for SSA therapy. Thirty-one patients entered the SSA group and 41 the AS group. The median interval between diagnosis and time from treatment start was 10.3 months. After controlling for potential confounders comparing the baseline characteristics among the cohorts, the two population were considered homogeneous, though the SSA group contained statistically significant more patients with larger tumors (*P* = 0.009). The baseline characteristics of patients are shown in Table 1.

**TABLE 1:** Patients' Clinicopathological Characteristics

Characteristics	SSA	AS	total	*P*
Number (%)	31 (43%)	41 (57%)	72	
Age (median; years)	66	64	64	0.482
Gender				1.0
Male	15 (48.3%)	21 (51.2%)	36 (50%)	
Female	16 (51.6%)	20 (48.7%)	36 (50%)	
Tumor site				0.367
Head	13 (41.9%)	12 (29.2%)	25 (34.7%)	
Body	11 (35.4%)	14 (34.1%)	25 (34.7%)	
Tail	7 (22.5%)	15 (36.5%)	22 (30.5%)	
Size				0.009
≤1 cm	9 (29%)	25 (60.9%)	34 (47.2%)	
>1 cm to 2 cm	22 (70%)	16 (39.1%)	38 (52.7%)	
Grade *				0.033
G1	21 (67.7%)	37 (90.2%)	58 (80.5%)	
G2	10 (33.3%)	4 (9.8%)	14 (19.5%)	
Ki67 **(**%**)**				0.178
1	11 (35.4%)	15 (36.5%)	26 (36.1%)	
2	9 (29%)	21 (51.2%)	30 (41.5%)	
3	3 (9.6%)	2 (4.8%)	5 (12.2%)	
4	1 (3.2%)	1 (2.4%)	2 (2.7%)	
5	4 (12.9%)	0 (0%)	4 (5.5%)	
6	1 (3.2%)	1 (2.4%)	2 (2.7%)	
7	1 (3.2%)	0 (0%)	1 (1.4%)	
10	1 (3.2%)	0 (0%)	2 (2.7%)	
Unknown	0 (0%)	1 (2.4%)	1 (1.4%)	
SSTR2 IHC†				0.658
0	0 (0%)	1 (2.4%)	1 (1.4%)	
1 +	0 (0%)	1 (2.4%)	1 (1.4%)	
2 +	1 (3.2%)	1 (2.4%)	2 (2.8%)	
3 +	29 (93.5%)	36 (87.8%)	65 (90.3%)	
Unknown	1 (3.2%)	1 (2.4%)	3 (4%)	

*According to WHO 2019 classification.^15^ †According to Volante score.^16^

### Patient Outcome

**FIGURE 2:**
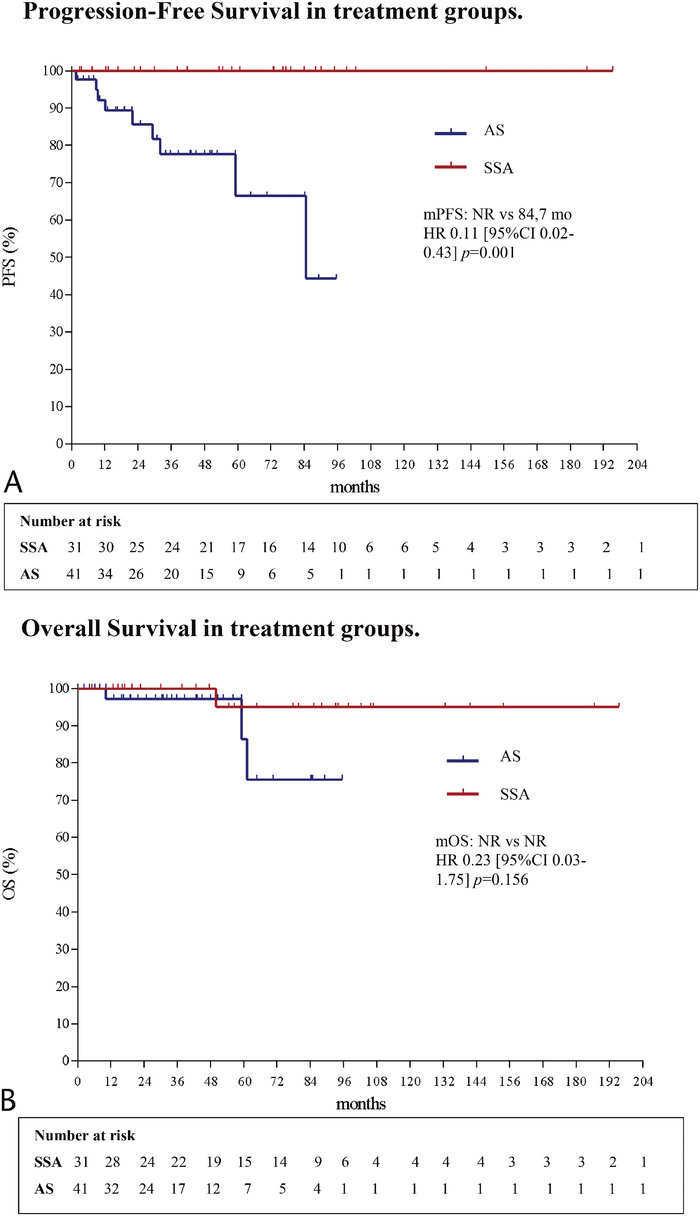
Survival outcomes. A, Progression-free survival in treatment groups. B, Overall survival in treatment groups.

At data cutoff analysis in April 2023, with a median follow-up of 53.7 months, no disease progression (PD) was reported in the SSA group, while the only event registered was related to patient death for reasons other than disease. Conversely, the PD or death rate was 19.5% (9 pts) in the AS group. In the SSA group the median PFS was not reached (NR), because of paucity of events, versus 84.7 months in the AS group (hazard ratio [HR], 0.11; 95% confidence interval [CI], 0.02–0.43; *P* = 0.001) (Fig. 2A). In the SSA group, the objective response rate (ORR) was 16.1% with 4 partial responses (12.9%) and 1 complete response (3.2%). In the AS group, 9 patients (21.9%) registered a progression disease or death event; specifically, in 66.6% of these patients, the primitive tumor progressed in its size, while 2 patients (22.2%) developed a metastatic disease. Patient with PD received SSA or surgery according to stage at progression, MTB decision, and patient preference. To note, in 2 patients (4.8%) in the AS cohort, death was directly related to disease progression. The median OS was NR in both groups (HR, 0.23 [95% CI, 0.03–1.75], *P* = 0.156) (Fig. 2B). An exploratory analysis was performed to evaluate the survival impact of demographic and clinicopathological factors. At Cox regression analysis, the baseline variables investigated (age, sex, tumor site, tumor size, stage, grade, and SSTR2 IHC expression) did not show any prognostic significance in the overall population nor influenced the benefits observed in the SSA group, suggesting the SSA treatment as the only variable impacting the outcome. The Cox regression analysis data for patients' variables are shown in Table 2. The rates of SSA-related adverse events are reported in Table 3.

**TABLE 2:** Cox Regression Analysis for PFS

Characteristics	*P*	HR	95% CI	
Age (<70 yo vs ≥70 yo)	0.245	1.04	0.97	1.11
Gender (F vs M)	0.143	5.29	0.56	49.41
Tumor site (head vs body vs tail)	0.342	1.61	0.59	4.37
Size (≤1 cm vs >1 cm)	0.849	0.98	0.87	1.12
Grade (1 vs 2)	0.857	0.76	0.03	15.06
Ki67%*	0.507	0.75	0.32	1.73
SSTR2A IHC†	0.561	0.59	0.10	3.46

*According to WHO 2019 classification.^15^ †According to Volante score.^16^

**TABLE 3:** Somatostatin analogs' Common Related Adverse Event Frequency in SSA Group (According to CTCAE Version 5.0)

	Grade 1	Grade 2	Grade 3	Grade 4	Grade 5
Diarrhea	**3 (9.7%)**	**1 (3.2%)**	**0**	**0**	**0**
Nausea	**0**	**0**	**0**	**-**	**-**
Bloating	**1 (3.2%)**	**0**	**-**	**-**	**-**
Cholelithiasis	**0**	**0**	**0**	**0**	**0**
Blood glucose changes					
Hyperglycemia	**1 (3.2%)**	**0**	**0**	**0**	**0**
Hypoglycemia	**0**	**0**	**0**	**0**	**0**
Sinus bradycardia	**0**	**0**	**0**	**0**	**0**
Injection-site reaction	**4 (12.9%)**	**0**	**0**	**0**	**0**
Fatigue	**0**	**0**	**1 (3.2%)**	**-**	**-**
Others	**0**	**0**	**0**	**0**	**0**

CTCAE indicates Common Terminology Criteria for Adverse Events.

## DISCUSSION

Despite PanNETs may be considered rare pancreatic neoplasms, accounting for about 2% of all pancreatic cancers,^20^ they are the second most common malignancy of the pancreas because their frequency almost doubled over the last 20 years.^21,22^ The great majority of newly diagnosed PanNETs are G1-G2 sporadic (not genetically determined), NF (not causing any syndrome due to hormone hypersecretion), lesions below 2 cm incidentally detected by the widespread use of axial imaging and/or endoscopic ultrasound in clinical practice for other reason. Whether a small NF, G1-G2 PanNETs requires treatment may depend on several factors, including both tumor's characteristics (eg, grade) and individual patient's circumstances. According to the European Neuroendocrine Tumor Society guidelines,^9^ if the PanNET is NF, not growing rapidly (G1-G2) and not causing symptom due to its localization in the pancreas (ie, in the head of the pancreas and likely to cause symptoms vs those in the body/tail), then it may not require surgery.^23^ Indeed, NF, G1-G2 PanNETs ≤2 cm are considered to have a lower aggressiveness and a better prognosis than larger tumors. Thus, recent studies have suggested that AS may be a viable option for certain patients with small, NF, G1-G2 PanNETs compared to surgery.^24,25^ However, data among literature are inconsistent and limited by the retrospective design of studies and the small number of patients investigated. Because of their relatively heterogeneous biology, the PanNET malignant potential and the possibility of progression are not negligible, particularly when considering their Ki-67 value and grade.^26–29^ Studies revealed lymph node involvement in up to 12% of NF, G1-G2 PanNETs ≤1 cm and in 26% of 1–2 cm.^30,31^ More recently, published data from an interim analysis of the prospective ASPEN trial revealed that a measurable tumor growth was observed in up to 51.0% of patients resulting in 14.1% of pancreatic resection after initial conservative treatment.^32^ Additionally, despite the usually excellent long-term outcome, even surgery harbors a 7.7% of recurrence rate.^26^ Of note, surgery is intrinsically burdened by significant morbidity and mortality. Kazanjian et al found that among 70 patients who underwent pancreatic surgery for PanNETs, postoperative complications occurred in 48.1% after pancreaticoduodenectomy and in 12.5% after distal pancreatectomy, respectively.^33^ The most common postoperative complication after pancreaticoduodenectomy for PanNETs is pancreatic fistula, which is even more frequently reported after enucleations than pancreatic resections accounting for more than half of complications.^34^ Despite the already known great efficacy of the SSAs in the advanced/metastatic setting, no evidence of the value of medical treatment with SSA exists for localized disease. The antiproliferative effect of SSAs was proven in advanced GEP-NETs by results from PROMID and CLARINET studies.^10–12^ Thus, offering SSA therapy seems to be an intriguing option for such patients diagnosed with small NF PanNETs. In our series, we offered the SSA therapy to patients with localized sporadic, NF, G1-G2 PanNETs ≤2 cm who had no indication for surgery and declined an AS program. After 16 years of enrolling patients and a median follow-up of 53.7 months, the SSA therapy emerged as a valuable strategy in our observational retrospective cohort study. In the SSA group, the median PFS was NR versus 84.7 months in AS group (HR, 0.11; *P* < 0.01). However, the SSA treatment reached a disease control rate of 100%, the only event registered was related to patient death for reasons other than disease. Moreover, in the SSA group, 4 patients reported a partial response (12.9%) and 1 reported a complete response (3.2%) according to RECIST 1.1 criteria with an overall response rate of 16.1%. Conversely, in the AS cohort, 21.9% of patients registered a progression disease. Taking a closer look to PD events, the majority (66.6%) experienced a size increase of the primitive tumor, above the >2-cm cutoff in 3 cases, in 1 patient with also the onset of hormone production related symptoms (diarrhea), which required SSA therapy, while 2 patients (22.2%) reported metastasis development (appearance of liver and nodal metastases). At last, 4 patients in the AS cohort started SSA therapy later on. Overall, in 2 patients (4.8%) in the AS cohort, death was directly related to disease progression. The rates of progression disease, distant metastasis, and disease-related death in AS cohort reported in this work were higher than expected when compared to the preceding PANDORA study^35^ and ASPEN trial^32^; the differences in follow-up strategy and tumor progression assessment criteria, together with the longer follow-up, may explain this discrepancy. At disease progression, our patients received SSA (5), surgery (1), or even continued AS (3), according to stage at progression, MTB indication, and patient's preference. The median overall survival was NR for both groups, because of relatively lack of events. Overall, in 2 patients (4.8%) in the AS cohort, death was directly related to disease metastatic progression and subsequent clinical worsening. Clinical-pathological characteristics such as sex, age, grading, tumor site, and dimension did not affect the benefits provided by SSA therapy. Our results suggest that in the unsolved dilemma between surgery or surveillance for small NF PanNETs might even be place for a third treatment option. In the SSA group, therapy was continued according to efficacy and tolerance. The optimal tolerability allows SSAs to be safely administered as long as the effect is observed, with a good compliance and in absence of significant toxicity and trifling impact on patients' quality of life. Indeed, only 10.3% of patient in SSA cohort reported mild drug-related toxicity, the most common (12.9%) were injection-site discomfort and erythema and diarrhea, usually lasting 24–48 hours after the SSA injection frequently after the first and second doses only. In 1 case after three doses, 1 patient decided to discontinue SSA because of fatigue (Table 3). However, there is little knowledge in literature about long-term use of SSA-related adverse events and the retrospective collection of safety data might not address this concern. Of note, for all patients in the SSA group, a prophylactic treatment with ursodeoxycholic acid was recommended to prevent cholelithiasis and related complications. In light of the above results, the decision to proceed with surveillance, surgery, or SSA treatment for a PanNET smaller than 2 cm should be tailored on a case-by-case basis, considering both disease aggressiveness and the individual patient's characteristics and preference.

The major limit of our study is the methodology of retrospective analyses itself, including selection bias and potential confounders. First of all, this work not included a third cohort of surgically resected PanNETs, thus, it is not possible to run any definitive conclusion about which one of these three option is preferable. To compare SSA against radical surgery as opposed to a watchful-wait strategy, prospective studies with larger sample size and longer follow-up are warranted. Given the recent proposition of SSA usage in such patients, it is plausible that AS was primarily favored for those enrolled in the study's early years. In addition, tumors diagnosed before 2010 might have been underestimated because of the delayed introduction of ^68^Ga-SSA PET/CT. To overcome this limit, we considered only patients who underwent at least one ^68^Ga-SSA PET/CT through their disease history. The relatively limited number of patients enrolled and the low rate of events may also have affected our statistical analysis. Finally, the employment of SSA can impose significant financial burdens, although this study was not designed to evaluate this issue.

In addition, consideration about SSA period of use, long-term safety, and criteria for completing treatment should be addressed prospectively.
